# Successful Discontinuation of Eculizumab in a Pediatric Patient With Atypical Hemolytic Uremic Syndrome and Underlying Systematic Lupus Erythematosus

**DOI:** 10.7759/cureus.25117

**Published:** 2022-05-18

**Authors:** Issa Alhamoud, Sydney A Freiberg

**Affiliations:** 1 Pediatric Nephrology, University of Iowa, Iowa City, USA

**Keywords:** case report, sle, systemic lupus erythematous, ahus, atypical hemolytic uremia syndrome, thrombotic microangiopathy, tma, eculizumab, pediatrics

## Abstract

Although rare, atypical hemolytic syndrome (aHUS) has been recognized as one of the direst complications of systemic lupus erythematosus (SLE). Furthermore, the diagnosis of coexisting aHUS and SLE is a diagnostic dilemma with similar clinical characteristics between both entities. Eculizumab is an effective treatment for complement-mediated atypical hemolytic uremic syndrome, but much is still to be learned about optimal treatment duration and if eculizumab can be discontinued without thrombotic microangiopathy reoccurrence. Here, we report a pediatric case of severe SLE complicated by aHUS that responded favorably to eculizumab, followed by successful discontinuation without recurrence of aHUS despite having numerous identified risk factors.

## Introduction

Systemic lupus erythematosus (SLE) is an autoimmune disease characterized by autoantibodies against self-antigens such as double-stranded DNA and nuclear antigens from overreactive B cells. One complication resulting from the autoantibodies is the formation and deposition of immune complexes in the small vessels of the kidney resulting in lupus nephritis (LN) [1]. The immune complexes occur when the autoantibodies (usually IgG) bind their antigen. At the same time, there is overactivation of the alternative complement pathway and the formation of numerous membrane attack complexes (C5b-9). Normally these complexes are cleared by red blood cells, but deficiencies in certain components of the classical complement pathway can inhibit this clearance [2]. It is this accumulation of complexes in LN patients that build up and damage the endothelium of the kidney’s blood vessels, termed thrombotic microangiopathy (TMA) [3]. The current recommendation for patients with SLE is treatment with immunosuppressive agents such as cyclophosphamide and mycophenolate mofetil, but the outcomes with these treatments alone are poor in the small subset (1-4%) of SLE patients that develop TMA [1]. 

Atypical hemolytic uremia syndrome (aHUS) is one form of TMA. It is a rare condition characterized by hemolytic anemia, thrombocytopenia, and acute kidney damage or failure. The characteristic symptoms of aHUS are due to microthrombi in small vessels throughout the body, especially the kidney glomeruli. Organ damage, high blood pressure, ischemia, and other serious complications highlight the importance of early detection and treatment. Development is often associated with secondary triggers such as infection, certain drugs, or autoantibodies, as well as other autoimmune conditions (such as SLE) [4]. Those with an increased risk of aHUS development often have excessive activation or dysregulation of the alternative complement pathway of the immune system. At least seven genes are associated with aHUS, many coding for complement regulatory components such as factor H, factor I, and membrane cofactor protein. Mutations making these regulatory components nonexistent or nonfunctional lead to an overactivation of complement and the characteristic signs of aHUS [5]. Only 50-60% of affected patients test genetically positive, however, and aHUS can still be diagnosed clinically. Steroids, immunosuppressants, and plasma exchange therapy removing autoantibodies and other harmful substances from the blood are standard treatments, but patients are still at risk for relapse and further kidney damage [4]. 

Eculizumab is a humanized anti-C5 monoclonal antibody approved by the U.S. Food and Drug Administration and has been shown to be a safe and effective treatment of aHUS, but much is unknown regarding optimal treatment duration and if eculizumab can be successfully discontinued without TMA reoccurrence [6,7]. One long-term observational study done by Menne et al. found that in patients with aHUS, discontinuing eculizumab therapy was associated with a decrease in renal function over time. Additionally, the study investigated risk factors for future TMA events after discontinuation of eculizumab treatment, finding that individuals with certain genetic, autoimmune, or complement abnormalities, and individuals with pediatric TMA onset were at a higher risk for future TMA events after discontinuation of treatment with eculizumab [8]. Although eculizumab has been shown to be safe for long-term use, patients are at increased risk for infection (especially meningococcal infections), hypertension, and infusion reactions. Additionally, side effects such as anemia, diarrhea, vomiting, peripheral edema, and upper respiratory infections; the additional burden of hospital visits for infusion sessions suggests there are benefits to discontinuing therapy [9]. 

Here we present a pediatric case of aHUS in a patient with severe SLE that responded favorably to eculizumab, followed by successful eculizumab discontinuation. Despite having numerous risk factors for relapse as identified in the Menne et al. study, this patient has not had another aHUS event since his initial event one year ago [8]. We then performed a brief review of the literature to see if eculizumab was successfully discontinued in other pediatric patients with aHUS/TMA and underlying SLE since aHUS/TMA is a rare occurrence in this small subset of at-risk patients and limited data is available for optimal eculizumab treatment duration in these patients.

## Case presentation

We herein report a case of an 18-year-old Caucasian male with a medical history significant for isodicentric 15q duplication with associated developmental delay, seizures/Lennox-Gastaut syndrome, neurobehavioral issues, attention deficit hyperactivity disorder, autistic behavior, and previously normal kidney function. At his initial presentation, he had experienced six months of weight loss, fatigue, and recurrent fever, and tested positive for antinuclear and anti-double-stranded deoxyribonucleic acid (dsDNA) antibodies in the presence of hypocomplementemia regarding his C3 and C4 levels. At that time, a bone marrow biopsy was negative for malignancy and hemophagocytic lymphohistiocytosis (HLH), so he was diagnosed with systemic lupus erythematosus. 

Two months after his diagnosis of systemic lupus erythematosus, he presented with acute kidney injury, recurrent seizures, hypertensive encephalopathy, worsening hemolytic anemia, and thrombocytopenia. After his manifestation of acute kidney injury with elevated serum creatinine levels (3.19 mg/dl from baseline 0.7 mg/dl), worsening proteinuria (urine protein/creatinine ratio of 2 mg/mg creatinine), and both worsening thrombocytopenia and hemolytic anemia, a kidney biopsy showed class IV lupus nephritis and severe lupus vasculopathy with features of thrombotic microangiopathy (TMA). His ADAMTS13 activity and inhibitor profile were normal. The thrombotic microangiopathy genetic panel (CFH, CFI, MCP, CFB, CFHR5, C3, THBD, DGKE, PLG, ADAMTS13, MMACHC, G6PD, and factor H autoantibodies) was also negative. 

He was treated with five doses of intravenous methylprednisolone (1 g/day) and intravenous cyclophosphamide (500 mg biweekly). This patient’s SLE was severe as suggested by his history of antiphospholipid antibody syndrome and necrosis of his toes due to Raynaud’s phenomenon that eventually required amputation.

**Figure 1:**
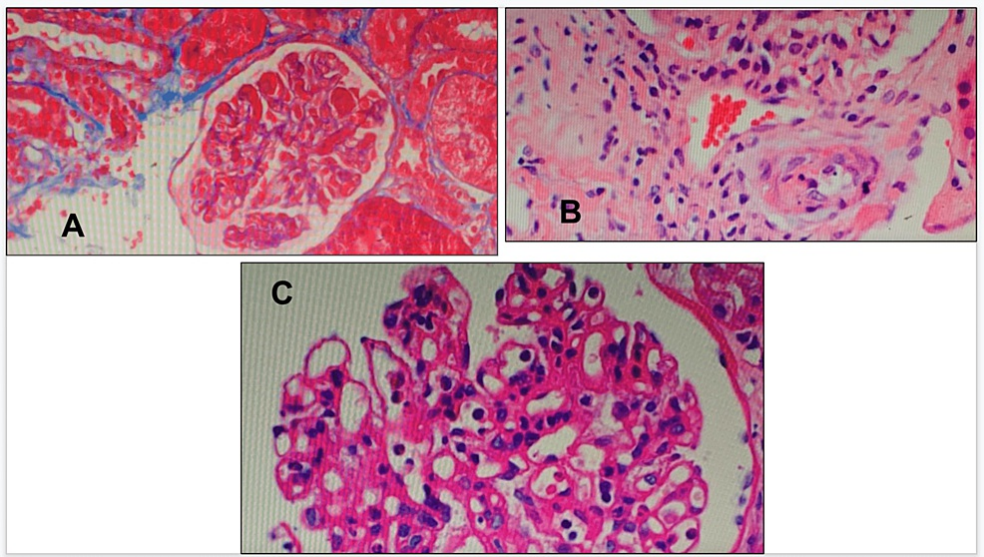
Kidney Biopsy Specimen (A) Fibrin thrombi (PAS: Periodic Acid-Schiff stain). (B) Arteriolar thrombotic microangiopathy (H&E: Hematoxylin & Eosin stain). (C) Proliferative glomerulus (JMS: Jones Methenamine Silver stain).

Two weeks later, a kidney biopsy was repeated due to worsening kidney function. The biopsy redemonstrated severe lupus vasculopathy and TMA, so he underwent five sessions of plasmapheresis over the span of one week (Figure 1). 

Due to oliguric acute kidney injury, continually worsening thrombocytopenia and hemolytic anemia, multi-drug resistant hypertension, and persistent hypocomplementemia even after three weeks of an extensive immunosuppressive regimen, an eculizumab regimen was initiated that resulted in a dramatic improvement of his clinic course. The eculizumab regimen consisted of 900 mg weekly for four weeks, then 1200 mg biweekly for six doses. In addition to eculizumab, he had received six doses of IV cyclophosphamide (500 mg biweekly), IV methylprednisolone (1 g weekly), prednisone (20 mg daily), and daily hydroxychloroquine. 

Within two weeks of initiating eculizumab, his serum creatinine improved from 3.19 mg/dl to 1.5 mg/dl with corresponding estimated glomerular filtration rate levels also improved from 22 to 47 ml/min/1.73 m2. Levels for complement C3, complement C4, hemoglobin, and platelet counts also improved as summarized in Table 1. 

Four months later, he continued to have stable serum creatinine levels, a normalized urine protein/creatinine ratio, a normalized complement regarding his C3 and C4 levels, improved hemoglobin, and a normalized platelet count. Because of his improved condition, the eculizumab regimen was discontinued and he continued a maintenance therapy consisting of daily mycophenolate mofetil, prednisone, and hydroxychloroquine for SLE.

**Table 1:** Laboratory Data Post Eculizumab Treatment

	Baseline	aHUS Event	Two Weeks Post Eculizumab	Four Months Post Event (Last Dose)	Six Months Post Event	Twelve Months Post Event	Reference Values
Creatinine (mg/dL)	0.7	3.19	1.5	1.45	1.18	1.1	0.6-1.2
Estimated Glomerular Filtration Rate (mL/min/1.73 m2)	101	22	47	49	60	64	>60
C3 (g/L)	40	40	68	79	90	98	90-180
C4 (g/L)	2	2	13	16	25	27	10-40
Hemoglobin (g/dL)	9.2	8.5	10	10	9.3	12.4	13-16
Platelets (per microliter)	101k	59k	124k	162k	157k	244 k	150-300k

## Discussion

Herein, we present a unique SLE case complicated by a severe TMA episode with a favorable response to eculizumab to the extent we were able to discontinue it with no relapse of TMA. This patient responded well to eculizumab during treatment, as demonstrated by his rapidly decreased serum creatine levels, normalization of complement, and increased eGFR. Four months after his initial diagnosis, the patient’s laboratory markers were improved to the point where he was stable. His complement values were approaching the normal range suggesting his SLE was under control and his improved estimated glomerular filtration rate and serum creatine levels indicated his kidneys were approaching near-normal function. Given these laboratory markers, the decision was made to discontinue eculizumab with a close follow-up. Further supporting this decision was his negative genetic panel which indicated the overactivation of the alternative complement pathway associated with aHUS was unlikely to be the result of permanent genetic factors making his aHUS would not be a reoccurring issue. In the months following discontinuation of therapy, his laboratory values are indications of his improving kidney function. Although this patient had numerous risk factors for future TMA events as identified in the Menne et al. study, he continues to remain in remission.

A brief review of the literature uncovered another pediatric case of SLE complicated by aHUS that was successfully treated with eculizumab. The search was performed on Ovid MEDLINE and EMBASE from 2010 to the present. The search terms used were “SLE AND TMA AND ECULIZUMAB” or “SYSTEMIC LUPUS ERYTHEMATOSUS AND TMA AND ECULIZUMAB” or “LUPUS NEPHRITIS AND ECULIZUMAB AND AHUS” or “LUPUS NEPHRITIS AND ECULIZUMAB AND TMA” or “SLE AND ECULIZUMAB AND AHUS”. Although the patient from the review had the same risk factors as our patient (being pediatric and having the underlying autoimmune disorder SLE) the patient from the review did relapse after discontinuation of eculizumab therapy. Like our patient, this patient received eculizumab therapy after other therapies such as cyclophosphamide, mycophenolate mofetil, plasma exchange, and rituximab were unsuccessful in controlling her lupus nephritis. After eculizumab treatment, her eGFR and proteinuria values returned to normal suggesting complete remission. Attempts to discontinue eculizumab were made twice, but the patient relapsed showing signs of kidney damage and aHUS both times. Restarting eculizumab therapy quickly controlled her aHUS/TMA in both relapse instances [10]. The results of this case from the review, congruent with the results of the Menne et al. study, seem to suggest that physicians should use caution before discontinuing eculizumab in this subset of patients [8]. The unique and conflicting results of our case, however, are reassuring, suggesting that eculizumab can successfully be discontinued without aHUS relapse given laboratory markers indicating adequate kidney function, stable SLE, and no apparent genetic causes for the aHUS event. Since reinitiating eculizumab therapy was effective in controlling reoccurring aHUS events in the case uncovered from the literature review, physicians might also be less hesitant to discontinue eculizumab knowing there are cases in which eculizumab was effective even after reinitiating treatment. Our review of the literature was limited, however, by the limited number of occurrences of aHUS/TMA in pediatric patients with underlying SLE, with even fewer of these patients having completed a full course of eculizumab treatment. Our case alone suggests that eculizumab can successfully be discontinued without relapse, but when combined with the case from the literature review and the results of the Menne et al. study, it demonstrates more research is needed to determine the optimal length of eculizumab therapy in these patients with multiple relapse risk factors [7,8].

Another unusual finding that makes our case significant is that our patient’s thrombotic microangiopathy genetic panel was negative. Half of aHUS patients test genetically negative, but our patient’s negative result was surprising given that the genetic screening also tested for aHUS-associated autoantibodies and our patient was experiencing an SLE flare at the time. In the case we identified from reviewing the literature, the patient also had signs of an SLE flare, demonstrated by low serum C3 and C4 and highly activated terminal complement. Surprisingly, however, this patient’s genetic evaluation for complement inhibitors was also negative. The genes investigated were more limited (CFH, CFI, MCP, THBD, CFB, and C3) and the only autoantibody test mentioned was for anti-ADAMTS13, however [10]. Considering both patients received negative genetic/autoantibody results, it suggests there could be another gene or autoantibody associated with SLE contributing to these patients' increased risk for aHUS development and relapse that was not tested for on routine panels. Studies such as these further suggest there are likely other antibodies involved in aHUS development, especially in patients with underlying autoimmune disorders such as SLE with negative genetic evaluations.

## Conclusions

Cases of aHUS in pediatric patients with underlying autoimmune disorders such as SLE are extremely rare, and the optimal length of eculizumab therapy in these patients with multiple relapse risk factors is not clear. Further complicating the optimal length of therapy in these patients is the absence or presence of certain genetic mutations and autoantibodies associated with aHUS. From our unique case, we learned that eculizumab can successfully be discontinued without aHUS relapse in a pediatric patient with underlying SLE given laboratory markers indicating adequate kidney function, stable SLE, and no apparent genetic causes for the aHUS event. Our case alone highlights that eculizumab can successfully be discontinued without relapse which is an interesting result as this would be an interesting area of multicenter research to determine the optimal length of eculizumab therapy in these patients with multiple relapse risk factors. A multi-center study would be helpful in capturing more of these rare cases and clarifying the mechanism of how SLE can trigger aHUS and increase relapse risk, especially in patients with negative genetic results. Even considering the potential complications and burdens associated with eculizumab therapy, the result of our case is reassuring in that eculizumab can be successfully discontinued without relapse despite numerous relapse risk factors.
